# A Conceptual Framework of Climate Action Needs of the Least Developed Party Countries of the Paris Agreement

**DOI:** 10.3390/ijerph19169941

**Published:** 2022-08-12

**Authors:** Usman Sattar

**Affiliations:** College of Law and Political Science, Zhejiang Normal University, Jinhua 321004, China; usman@zjnu.edu.cn

**Keywords:** climate governance, net-zero carbon emissions, climate justice, climate finance, law and policy, technology, mitigation and adaptation, education, environment, sustainability transitions

## Abstract

This article provides a framework for conceptualizing climate action needs grounded in the nationally determined contributions (NDCs) of the least developed party countries (LDPCs) of the Paris Agreement (PA). It examines the NDCs of 35 LDPCs recorded in the NDC public registry of the United Nations Framework Convention for Climate Change (UNFCCC). A grounded theory approach is adopted to assess what these countries need to materialize their NDCs under the PA. A conceptual framework of needs is figured out through an iterative process of data collection and analysis in three cycles: (1) open and in vivo coding; (2) axial coding; and (3) theoretical or selective coding. The data are analyzed with the help of NVIVO software. The results provide a verifiable framework of needs for climate action, which includes 55 saturated need factors extracted from the writing excerpts of NDCs, 17 sub-categories (axial codes) with climate finance and technology transfer as the most prominent, and 7 theoretical or selective categories with mobilize, educate, governmental, synergic, levels, equity, and public health. It provides a baseline for policy, research, and action from the developed party countries to uphold their PA obligations.

## 1. Introduction

Climate change, with its continuous increase in greenhouse gas (GHG) emissions, is now disrupting lives everywhere [1]. Despite their nominal contribution to GHG emissions, the least developed countries are the most vulnerable to its consequences [2,3,4]. It raises concerns related to climate justice and climate governance [5]. To tackle the issue, the United Nations (UN) under Goal 13 of the Sustainable Development Goals (SGDs)—Climate Action—175 countries initially signed the PA after the 21st conference of parties (COP21) in Paris in December 2015 [6]. Today, 193 countries—almost all countries on earth—have joined the PA. Articles 2 and 3 of the PA require all party countries to “undertake and communicate” (p. 3) their NDCs in order “to hold the increase in the global average temperature to well below 2 °C above pre-industrial levels and pursuing efforts to limit the temperature increase to 1.5 °C above pre-industrial levels” (p. 3).

The Paris Agreement refers to the party countries in three categories: developed party countries, developing party countries, and the least developed party countries. Under Article 4 of the PA, each party country is free to decide its own “common but differentiated responsibilities” (p. 6) according to their respective capabilities and national circumstances. Developed party countries, under Articles 9, 10, and 11 of the PA, are obliged to provide support to the developing party countries, and especially to those parties who are particularly vulnerable to the adverse effects of climate change, such as the least developed countries.

To achieve the goal of the PA by the end of this century (in the long run), at COP26 in Glasgow last year (2021), all party countries stressed the need to reduce carbon dioxide (CO2) emissions by 45 percent in this decade (by 2030) and net zero emissions by 2050 (in the short run). It requires accelerated action for emission reduction from all party countries and leveling up support from the developed countries to the developing and least developed countries. All party countries have submitted their initial NDC targets where the LDPCs have informed their needs under Articles 9, 10, and 11 of the PA.

The focus here is on the types of needs that the poorest and most vulnerable countries to climate change impacts have addressed in their NDC submissions. What specific support do they require or rely on from the developed world in order to transition from business as usual (BAU) to carbon neutral development in LDPCs? For instance, what kind of technology do they need to safeguard their countries from climate risks? What is the hierarchy of needs, and are there any shared or common needs where the LDPCs are looking for support from the developed world? In a nutshell, what are their climate action needs to achieve their NDC targets for climate risk reduction and GHG emission reduction for sustainability transitions?

A multilevel perspective (MLP) for socio-technical transitions [7] offers a tentative answer here. It provides three analytical levels—niche innovations, socio-technical regimes, and socio-technical landscapes—with multiple subcomponents at each level in order to avoid path dependency (BAU scenario) for sustainability transitions; however, three analytical levels appear to be operational for local governance [8,9] and lack operationalization on the LDPCs. Similarly, a socio-institutional approach to understanding the complex systematic needs for sustainability transitions also focuses on sectoral issues such as agriculture, industry, finance, business, education, or energy, and places limits on a particular geographical location [10,11,12,13,14,15,16,17]; the scope of this study is beyond the available approaches in the literature. It includes various regions, including African, Asian, and Island countries.

Therefore, it appears that, despite different disciplines having framed needs for climate change adaptation or developing preconditions for sustainability transitions, a consistent framework, particularly focusing on analyzing the climate action needs of the LDPCs, is not available. This article studies the NDC submissions of 35 LDPCs recorded to the NDC public registry of the UNFCCC. The UN [18] reports that the socio-economic outlook of these countries is grim. They accounted for just 0.13% of global trade in the 2010s, and 85% of these are commodity dependent. In 2020, they reported the worst growth rate in the last three decades; however, Bangladesh, with the trade liberalization policies of 1990, led to a boom in exports and is expected to no longer be a least developed country in 2026 [19].

Keeping in view the socio-economic circumstances of the LDPCs, international support with respect to climate action in these countries is crucial. This article identifies the climate action needs of these countries in order to frame their diverse needs into a conceptual framework for research, policy making, and response from the developed world; thus, it clearly aims to provide a strategic orientation to those countries that are obliged to assist LDPCs under Articles 9, 10, and 11 of the PA.

To the best of the author’s knowledge, this article is the first of its type that studies NDC submissions and provides an analytical model of needs grounded in the writing excerpts of NDCs. This framework offers a starting point for research on different needs components and their relationships defined in it. It also formulates an operational scheme to assist the UNFCCC, the Intergovernmental Panel on Climate Change (IPCC), and climate action (Goal 13 of the SDGs) related stakeholders at the national, regional, and international levels to reflect on the spectrum of NDC implementation conditions of the LDPCs. It also assists the least developed countries in updating their NDC submissions and reporting on the status of implementation in a systematic way. It can further assist the upcoming COP27 with policymaking on the LDPCs.

## 2. Materials and Methods

### 2.1. Approach

A grounded theory approach initially developed by sociologists [20,21] is adopted to develop a codes-to-theory model [22] (pp. 14–15) of needs grounded in the NDC submissions of the LDPCs of the PA. This is an inductive research approach that provides a step-by-step guide for theory development [23]. It has widely been used across disciplines, including social sciences [24], medical sciences [25], and business studies [26], and is now being used in environmental psychology [20], environmental sociology [27], and sustainability studies [28,29].

### 2.2. Data

To identify the needs of the LDPCs, a recently published (2021) list of 46 countries declared as the least developed countries in the post-COVID world by the United Nations Conference on Trade and Development (UNCTAD) [18] is reviewed to check their status as party countries to the PA. All the 46 least developed countries listed in the list are found to be a party to the PA. The NDC submission records of these 46 LDPCs were accessed from the NDC public registry of the UNFCCC. Out of 46 countries, 11 NDCs were found to be submitted in languages other than English. The conception of needs in this study is delimited to the data set of 35 LDPCs whose NDCs were available in the English language.

### 2.3. Data Analysis

**Figure 1:**
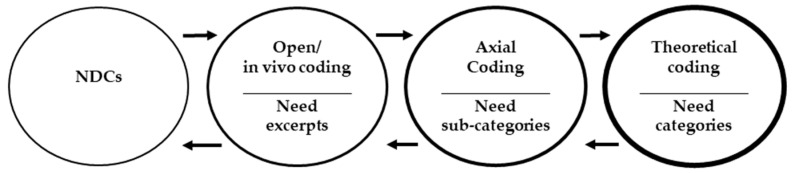
Iterative process of identifying needs and coding them into categories.

The data analysis is guided by [22] theoretical coding (p. 250), axial coding (p. 244), and open or initial coding (p. 110) with a mix of in vivo coding (p. 105) methods followed by three cycles of analysis. In the first cycle analysis, the needs communicated in NDCs are divided into discrete need patterns following open coding, and specific words or phrases specifying emphasis or critical needs are retained following in vivo coding. The variety of 55 discrete need factors/patterns is then harmonized into 17 axial codes (sub-categories) in the second cycle analysis. In the third cycle of analysis, 17 sub-categories are then aggregated into 7 key categories that structure the conceptual framework of grounded needs guided by theoretical coding. Finally, to facilitate a conceptual framework of needs, 55 need factors and 17 sub-categories of needs are aggregated into 7 key categories. The coding process remained iterative at each stage of analysis (Figure 1) and was validated by independent reviewers in a focus group discussion moderated by the author. All the need factors, sub-categories, and categories are discussed one by one to confirm the appropriateness of the wording used in given codes and their relationships and interdependence. NVIVO software is used to code and analyze the data.

### 2.4. Trustworthiness

To meet the trustworthiness of the saturated strands (needs) of this framework, this paper follows the criteria guided by [30], (1) familiarizing with the data; (2) generating initial codes; (3) searching for broader categories; (4) reviewing categories; (5) defining and naming categories. This framework can be tested with further research. It offers an action-oriented and trustworthy scheme for policy and research.

## 3. Results

Climate Action Needs (CAN): Their Relationships and Interdependence.

**Figure 2:**
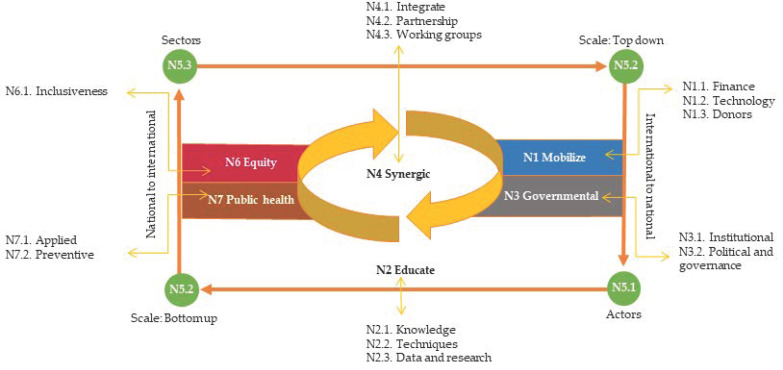
CAN model: overview of need categories, their relationships, and interdependence.

The CAN framework suggests 7 interdependent key categories of needs (theoretical codes), 17 sub-categories (axial codes), and 55 factors of need (initial and in vivo codes). An overview of the 7 interdependent key categories with their sub-categories is provided in Figure 2. The description of the retrieved sub-categories of needs starts from Section 3.1.1 Finance (N1.1).

### 3.1. Mobilize

#### 3.1.1. Finance (N1.1)

Seeking climate finance is the most coded/referred factor in NDCs. All the LDPCs have urged financial assistance, mainly from the international community (N1.3). They have conditioned most of their NDC targets with international financial support. They need it for the capacity building of different sectors (N5.3) and preventive measures (N7.2) against climate change risks. They wish to have very “timely” (Solomon Island, p. 20) and “direct access” to these funds under the “readiness” program of the Green Climate Fund without any intermediary agencies (Kiribati, pp. 24–25). They need climate finance in the form of “aid,” “concessions” (Sao TEP, p. 3), or “grants” (Chad, p. 11) as they contribute a very small proportion of GHG emissions yet are highly vulnerable to climate risks. Gambia (p. 28) urges adjustment in the support architecture by giving priority to the programs that generate strong domestic efforts, such as climate finance.

#### 3.1.2. Technology (N1.2)

Technology is the second most coded (needed) component after finance. The LDPCs need technologies for preparedness against climate change. This includes, but is not limited to, “early warning systems”, “smart agriculture technologies”, “efficient-energy”, and flood-resistant infrastructure (Eritrea p. 25, Kiribati p. 3, South Sudan p. 145). With this technology transfer, they also require knowledge (N2.1) and techniques (N2.2) to utilize them (Lesotho and Burundi). This process of technology transfer, as Angola notes, requires partnership (4.2) and institutional (N3.1) arrangements. Djibouti (p. 14) acknowledges its partnership with Germany and technological support in the energy sector for renewable energies (N5.3). 

#### 3.1.3. Donors/funders (N1.3)

The LDPCs, including Gambia, Sudan, and Nepal, have particularly appealed to the UNFCCC and the World Bank to ensure funding through the Green Climate Fund (GCF), the Least Developed Countries Fund (LDCF), the Special Climate Change Fund (SCCF), Climate Investment Funds (CIFs), the EU Global Climate Change Alliance Programme, the Scaling up Renewable Energy in Low Income Countries Programme (SREP), and more. They need funding from different actors (N5.1) for different sectors (5.3) at different scales (N5.2). 

**Figure 3:**
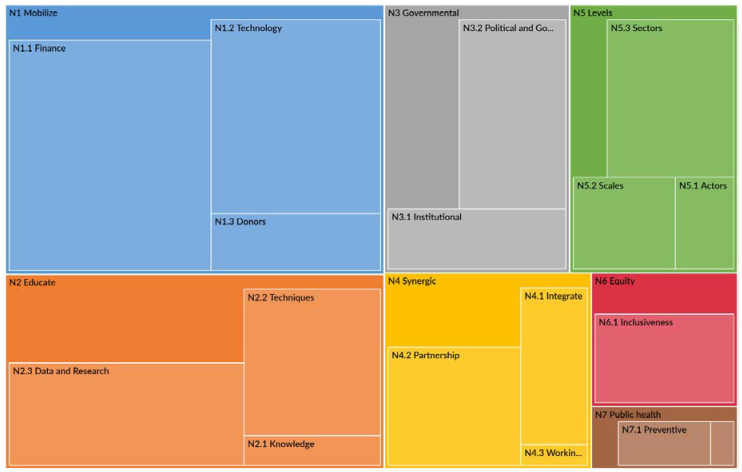
Hierarchy of needs: large area indicates more coding references from NDCs.

The three aforementioned sub-categories (N1.1, N1.2, and N1.3) and all others have some hierarchy based on the references taken from the NDCs. A detailed hierarchy chart of need categories and their sub-categories is provided in Figure 3. 

### 3.2. Educate

#### 3.2.1. Knowledge (N2.1)

Angola mentions that climate action requires sharing scientific knowledge and increasing qualified human resources. In general, LDPCs are short of qualified people who have the know-how about climate change issues. They need formal and informal education and public awareness programs. They need study and training abroad programs.

Kiribati and Burundi urge communicating climate knowledge to the general public by translating it in a way that the local people are able to relate it to their religious beliefs, traditional knowledge, and cultural practices. Similarly, Myanmar, Solomon Islands, and Malawi insist on sharing, documenting, and distributing adaptation research (N2.3) knowledge with them. 

#### 3.2.2. Techniques (N2.2)

Financial assistance and technology transfer alone are not sufficient for the implementation of NDC in these countries. They need skills and training for their staff and stakeholders to enable them to carry out technical assessments and operations related to NDC implementation (Benin, Uganda, and Burundi). They need technical assistance in different sectors (N5.3). For instance, Burundi, Rwanda, Lao DPR, and Liberia referred to climate risk screening, early warning systems, and procedural matters related to planning, budgeting, and administration of gaining climate finance (N1.1). The procedures involved in applying for grants (N1.1, N1.3), etc., and the identification of climate action needs and “differentiated” (PA) action plans depend upon international technical assistance. Bhutan needs techniques for robust forest monitoring; Burundi needs to learn the technical processes involved in the sustainable production of new crops; Myanmar requires assistance in identifying their country’s needs (N2.3).

#### 3.2.3. Data and Research (N.2.3)

Eritrea urges that there is a “dire need” for climatic databases for research. Similarly, Tuvalu and Lesotho have endorsed the same need. Others, including Chad, Kiribati, and Sao TEP, need study teams capable (N2.2) of vulnerability analysis, climate risk mapping, and delivering NDC targets (Tanzania). Most of the LDPCs, including Angola and Guinea Bissau, have reported the absence of local climate data and a lack of expertise (N2.2) in the data collection. Uganda requires studies on smart and sustainable agriculture practices according to the local climate infrastructure.

### 3.3. Governmental

#### 3.3.1. Institutional (N3.1)

LDPCs need institutional frameworks to achieve their NDC (Angola, Sao TEP). Lao PDR, Lesotho, and the Solomon Islands require legislation to strengthen mitigation and adaptation initiatives and integrate (N4.1) them into their development plans. Chad, Burundi, and Guinea Bissau need assistance in institutionalizing a sector-wise (N5.3) adaptation plan to safeguard natural resources. Mozambique reports that these institutional arrangements require financial assistance (N1.1) and Nepal requires to “enact key acts and regulations” to facilitate NDC implementation.

#### 3.3.2. Political and Governance (N3.2)

The LDPCs have political and governance challenges in implementing their NDCs. Sudan reports political sanctions by some developed countries, which have restricted the country’s access to bilateral climate finance. It has restricted its ability to contribute under the PA. Political instability in a country creates a leadership gap and governance issues. Guinea-Bissau and the Central African Republic state the need for political stability and an effective governance/policy structure where all the stakeholders (N5.1) join together in a supra-party manner for climate action. Benin suggests regulations on the international import of carbon-intensive electronic equipment. Uganda, Mozambique, and Lao PDR need to strengthen their ability to regulate, integrate (N4.1), and implicate climate policies.

### 3.4. Synergic

#### 3.4.1. Integrate (N4.1)

Burundi needs integration of climate resilience and response measures into all levels (N5) of development plans. To facilitate the operational measures of NDCs, the Central African Republic and Guinea Bissau have urged all the stakeholders to unite in front of climate change and take mitigation and adaptation steps without any jurisdictional conflicts and in a timely manner. Eritrea urges the integration of adaptation measures and synchronizing of incongruent data (N2.3) and working systems of different working groups (N4.3) among the various entities of national institutions (N3.1).

The need factors taken from each country is provided in Table 1, and their shared needs are grouped into sub-categories in Table 2. And, a consistent framework of 55 shared need factors with their sub-categories and categories is provided in Table 3. 

#### 3.4.2. Partnership (N4.2)

LDPCs require partnerships at different levels of scale (5.2) and between actors (5.1) and sectors (5.3). Angola mentions that bilateral and multilateral agreements on financial (N1.1) and technical (N2.2) support are “essential” for the further development of communication, public mobilization, and adaptation actions. Similarly, Djibouti and Uganda need national and international partnerships with university centers (N2.3) and private companies for technological advancement (N1.2) in the energy sector (N5.1).

#### 3.4.3. Working Groups (N4.3)

Lesotho and Rwanda have urged a systematic working environment where different working groups at different levels (N5) not only plan their transitions together but also design their implementation and follow-up adaptation practices. It will help LDCs learn from each other’s experiences and move jointly to achieve their NDC targets.

### 3.5. Levels

#### 3.5.1. Actors (N5.1)

Actors in steering positions are important players in NDCs. These players range from individual to organizational, institutional, and systematic levels. The Gambia and Guinea Bissau, among others, require the capacity building of these actors with financial (N1.1) and technical (N2.2) assistance according to Article 6 of the Paris Agreement.

#### 3.5.2. Scale (N5.2)

The LDPCs, including Gambia, Nepal, the Solomon Islands, and Guinea Bissau, have urged the capacity building of the actors (N5.1) at different scales. This includes local to city level support and from provincial to subnational, national level, and international level (N1.3) assistance. These scales are helpful in dividing responsibilities for the actors at different levels.

#### 3.5.3. Sectoral (N5.3)

Tuvalu needs standby diesel (fossil fuels) to generate renewable energy. Requiring fossil fuels for NDCs under the PA seems different but shows that not only in the energy sector but also in other sectors, sectoral transitions might require carbon-intensive initiatives to fuel sustainability transitions. Guinea Bissau, Gambia, and Lao PDR have urged financial and technical (N1.1, N1.2) support for the following sectors, but not limited to: health, renewable energy, lands, oceans, and coastal zone management, agriculture, livestock, environment, transport, forestry, fisheries, socio-economy, and education (N2.1, N2.2).

### 3.6. Equity, Equality, and Climate Justice

#### Inclusiveness (N6.1)

Solomon Islands, Burundi, Nepal, Uganda, and Guinea-Bissau have been urged to consider gender, youth, and vulnerable groups in disaster risk assessments and climate action plans. The least developed countries have a very small contribution to GHG emissions yet are at the frontline of the wrath of sea-level rise and climate change. The Central African Republic and Chad emphasize interregional socio-economic equality, human rights, and gender equality in meeting the PA goal. Kiribati (p. 27) and the Solomon Islands (p. 21), two of the smallest contributors to GHG emissions, call their NDC a “moral imperative” and urge the major carbon emitters to “drastically and immediately” reduce their emissions under the PA.

### 3.7. Public Health

#### 3.7.1. Applied Health (N7.1)

LDPCs face insufficient health care facilities in general and climate-resilient health facilities in particular. Myanmar needs intensive care units for treating heat-related and other climate change health disorders. Similarly, the Central African Republic needs preventive measures for waterborne diseases and seasonal pathologies. Solomon Islands urges the effective elimination or control of COVID-19 transmission as it affects the implementation of NDCs.

#### 3.7.2. Preventive Health (N7.2)

LDPCs in general and coastal areas, in particular, are in dire need of preventive health measures against the rise of sea levels. Burundi and Lesotho report their poor capacity structures to prepare for climate disasters and urge the international community for assistance in protecting their socio-economic systems (N1.1) against climate change vulnerabilities and enhancing their adaptive capacity for interpreting, communicating, and guiding local communities to deal with climate change.

## 4. Conclusions

This article started by pointing out the questions relating to what might enable the LDPCs to contribute their part in meeting the PA target. Given the LDPCs’ needs to materialize their NDCs, it appears that these countries are far behind in meeting the level of urgency PA requires to meet its carbon neutrality targets both in the short and long run. A critical review of 55 need factors, 17 sub-categories, and 7 core interdependent categories—CAN framework grounded in NDCs—reveals that the LDPCs are not simply requiring finance and technology but other complex and time-consuming needs as well. Their needs range from basic climate knowledge to skills and techniques, starting from the level of applying for grants to utilize them effectively. It also includes cultural interventions and religious affairs. They also pointed out equitable and just development at the local, regional, and global levels.

Interestingly, public health is the least coded category among all the needs categories. It shows fewer coding references related to health in their NDC submissions. Is it fair to consider health the least important component to mention in NDCs in the climate change scenario? It might be the most desperately needed category worth mentioning in NDCs, with synergic needs such as climate finance and technology; however, this study objectively visualizes the hierarchy of need strands according to the number of references/codes available in the NDCs. Another critical viewpoint is that the countries that are requiring assistance in capacity-building need assessment due to a lack of technical expertise, so on what basis are they requiring climate funds if they do not have the expertise to assess their needs or to utilize the required funds for the purpose they are looking for?

This article provides a first conceptual step towards understanding LDPCs’ needs in a coherent framework for policymaking, research, and global response under the PA. It appears to be a challenging task for the developed countries to meet LDPCs’ needs as per their commitments under Articles 9, 10, and 11 of the PA. This article focuses on the needs of the least developed countries by analyzing their NDC submissions. In the next step, looking into the needs of developing countries can further contribute to the area of research. Hence, looking into the NDCs of the developed countries will further illuminate how far and to what level they are able to enable LDPCs and developing countries to implement their ambitious targets to meet the goal of the PA.

Finally, NDCs are the pulse of climate change mitigation and adaptation, net-zero carbon emissions, and sustainability transitions. The global NDCs will determine how far countries are ambitious to deviate from the BAU scenario to meet the target under Article 2 of the PA and achieve Goal 13 of the SDGs. Climate governors must look beyond moral imperatives and see carbon neutrality as absolutely necessary for leapfrogging the carbon-intensive development paths around the world [31]. In addition, it has to be inclusive by bringing all parties together, similar to a family (countries) and home (earth). The developed countries under the upcoming COP27 negotiations may consider funding to organize a step-by-step training session for stakeholders and staff from the LDPCs in order to enable them to meet their NDC targets, achieve net-zero emissions, and safeguard their countries from climate change risks.

## Figures and Tables

**Table 1:** Need (N) factors communicated in the NDCs.

Country	Date	N1			N2			N3		N4			N5			N6	N7	
		1	2	3	1	2	3	1	2	1	2	3	1	2	3	1	1	2
Afghanistan	2015	●	●				●	●						●	●			
Angola	2021	●	●	●	●	●	●	●	●		●	●	●	●	●			
Bangladesh	2021	●	●		●	●	●			●			●	●				
Benin	2021	●	●			●	●	●	●	●				●	●			●
Bhutan	2021	●		●	●	●	●	●					●	●	●			
Burkina F.	2021	●					●				●		●	●	●			
Burundi	2015	●	●		●	●	●	●	●	●			●	●		●		●
Cambodia	2020	●		●		●	●	●		●	●		●					
Central A.R.	2015	●	●	●	●			●	●	●					●	●		●
Chad	2015	●			●		●	●	●	●		●				●		
Djibouti	2015	●	●								●			●				●
Eritrea	2018	●	●		●		●	●		●	●			●	●	●		
Ethiopia	2021	●	●					●					●	●	●			
Gambia	2021	●	●	●		●	●	●	●	●			●	●	●			
Guinea B.	2021	●	●			●		●	●	●	●		●	●	●	●		
Kiribati	2016	●	●		●		●	●		●	●				●	●		
Lao P.D.R.	2021	●	●			●		●	●						●			
Lesotho	2017	●	●			●	●	●			●	●						●
Liberia	2021	●	●			●		●			●	●			●			
Malawi	2021	●	●		●	●					●	●						
Mozambique	2021	●				●		●	●				●					
Myanmar	2021	●	●		●	●	●			●							●	
Nepal	2021	●		●			●	●	●	●		●		●		●		
Rwanda	2020	●	●			●	●			●		●						
Sao T.E.P.	2021	●					●	●										
Sierra L.	2021	●								●		●						
Solomon I.	2021	●	●		●		●	●			●			●		●	●	
Somalia	2021	●																
South S.	2021	●	●		●	●	●	●	●						●			
Sudan	2021	●		●					●									
Tanzania	2021	●				●	●											
Timor-Leste	2016	●	●	●							●				●	●		
Tuvalu	2015	●					●								●			
Uganda	2021	●	●			●	●		●		●				●	●		
Zambia	2021	●	●															

**Table 2:** Need factors grouped into sub-categories.

N1.1	Finance	N4.2	Partnership/exchange/coordinate
N1.2	Technology	N4.3	Working groups
N1.3	Donors	N5.1	Actors
N2.1	Knowledge	N5.2	Scale
N2.2	Techniques	N5.3	Sectoral
N2.3	Data and research	N6.1	Equity, equality and climate justice
N3.1	Institutional	N7.1	Applied health
N3.2	Political and governance	N7.2	Preventive health
N4.1	Integrate		

**Table 3:** A conceptual framework of climate action needs.

*Category,* Sub-Category, and Key Need Factors	Country NDC (Page no.) ^1^
***N1 Mobilize*** **N1.1 Finance**	
All party countries need financial support. Some parties have clarified that this support is required in the form of “aid,” “grants,” or “concessions.”	Chad, (11) Sao TEP. (3)
“Timely” and “direct access” to funds under the “readiness” program of the Green Climate Fund (GCF) without depending upon intermediary agencies.	Solomon I. (20) Kiribati (24, 25)
Adjusting financial architecture by giving priority to the programs that generate strong domestic efforts and designing disaster relief/insurance facilities.	Gambia (28)
**N1.2 Technology**	
“Climate smart agriculture” technologies (E), “fresh ground water lens” (K), “grid-connected photovoltaic system” (K), “early warning systems”, “energy-efficient cooking stoves” (S), and technology for a flood-proof infrastructure.	Eritrea (25) Kiribati (3, 24) South S. (58)
Technology transfer includes not only tools or equipment but also the expertise, skills, and technical knowledge required to utilize them.	Lesotho (10) Burundi (11)
**N1.3 Donors**	
The Least Developed Countries Fund (LDCF) and the Special Climate Change Fund (SCCF) of the UNFCCC; the GCF; EU Global Climate Change Alliance Programme; Climate Investment Funds (CIFs) of the World Bank; the Scaling up Renewable Energy in Low Income Countries Programme (SREP) and more.	Gambia (27) Sudan (16) Nepal (19, 20)
***N2 Educate*** **N2.1 Knowledge**	
Climate change response necessitates technical and human resource expertise. Qualified human resource for the development and implementation of NDC targets.	Angola (61)
Translating climate science and predicted impacts into messages that people can relate to cultural practices, traditional knowledge, and religious beliefs.	Kiribati (20)
To strengthen climate resilience and response education and integrate sustainability principles into formal education. “Documenting and distributing” (M) knowledge on climate change.	Myanmar (46) South S. (42) Burundi (7)
Sharing adaptation knowledge and increasing public awareness about climate change adaptation and disaster risk reduction.	Solomon I. (2) Malawi (8)
**N2.2 Techniques**	
Technical skills and human resource expertise for climate change response.	Benin (25)
Training stakeholders, medical pyramids, NDC implementing entities, and executives to deal with the harmful effects of climate change.	Burundi (11) Guinea B. (21)
Developing systems of information for climate risk/flood warning and monitoring the progress.	Rwanda (19) Lao DPR (8)
Technical operations for climate risk screening, budgeting, administration, and policy design.	Liberia (4, 34)
To determine the cost of the losses and damage caused by climate change.	Mozambique (15)
Robust forest monitoring system.	Bhutan (6)
Technical process involved in the sustainable production of new crops.	Burundi, (4)
“Technical assistance in identifying specific climate actions that can be used to mobilize international climate finance for meeting own NDC targets”.	Myanmar (52)
**N.2.3 Data and Research**	
There is a “dire need” (E) for obtaining and harmonizing climatic databases for research and climate action.	Eritrea (25)
Setting up study teams and strengthening their capabilities for the collection and analysis of local climate data.	Lesotho (10) Kiribati (21)
“Research on the vulnerability and adaptation of socio-economic sectors to climate change.”	Sao TEP (1) Burundi (12)
Vulnerability analysis, risk mapping, and a robust data collection system. Researchers for the effective delivery of intended output (NDCs target).	Angola (81) Tanzania (21)
***N3 Governmental*** **N3.1 Institutional**	
Legislation on mainstreaming climate change issues into development plans and revising environmental laws accordingly.	Lao PDR (13) Solomon I. (18)
Institutional arrangements in order to achieve the NDCs target. Defining institutional priorities for a sector-wise adaptation plan.	Angola (32) Chad (6)
“Institutional component is the biggest challenge.” In need of capacity building for the implementation of NDCs, monitoring, and production of GHG inventories according to the rules defined by the IPCC.	Guinea B. (34)
Institutional capacity building requires international climate action. Strengthening capabilities for consolidating institutional frameworks.	Mozambique (55) Sao TEP (1)
Institutional measures to safeguard the atmosphere, land, forest, oceans, and water resources. “Enact key acts and regulations” to facilitate NDC implementation.	Burundi (12) Nepal (20)
**N3.2 Political and Governance**	
Accessibility to bilateral climate finance is restricted due to political sanctions by some developed countries.	Sudan (16)
Political stability is significant in implementing NDCs.	Guinea B. (9) Central AR. (14)
Government and local agents are required to work together in a supra-party manner with a minimum governance structure on climate adaptation actions.	Guinea B. (34)
Effective regulations and control on the import of electronic equipment and promoting energy efficient operations.	Benin (31)
Create/improve public policies for the implementation and adaptation actions.	Guinea B (28)
Ability to enforce climate laws and regulations and strengthening cross-sectoral coordination for integrating them into policies.	Mozambique (25) Lao PDR (1,2)
***N4 Synergic*** **N4.1 Integrate**	
Integrating climate issues into development plans and policies.	Burundi (7)
One window operation for mitigation and adaptation measures in a way that all the concerned stakeholders are on a single page for action without any jurisdictional conflicts and delays.	Guinea B. (35) Central AR (14)
Synchronize incongruent data and systems among the various entities in the national institutions.	Eritrea (26)
**N4.2 Partnership** Developing international partnerships. The involvement of key players in the development of communication channels and public mobilization through bilateral and multilateral agreements is “essential”.	Angola (65)
Forming international and national-provincial alliances with university centers and private companies capable of providing technologies such as geothermal, wind, and photovoltaic power plants.	Djibouti (5, 6)
**N4.3 Working Groups**	
To establish a systematic working environment in which working groups from various sectors bring development partners, governments, private sectors, and civil society together to follow up on implementation plans.	Lesotho (26) Rwanda (70)
***N5 Level(s)*** **N5.1 Actors**	
Individual, organizational, institutional, and systematic levels. Education: primary, secondary, tertiary, and higher.	Gambia (33) Mozambique (67)
“Capacity building of actors to take advantage of carbon market mechanism as provided by Article 6 of the Paris Agreement”.	Guinea B. (21)
**N5.2 Scales** (Top-down and bottom up)	
“Scaling up climate action” (N) at community (local), city, provincial, subnational, national, and international levels.	Guinea B. (12) Nepal (20) Solomon I. (17)
**N5.3 Sectoral**	
Health, renewable energy, land, oceans and coastal zone management, agriculture, livestock, environment, transport, forestry, fisheries, socio-economic, and education.	Guinea B. (21) Gambia (22)
To generate 100% renewable energy by 2020, Tuvalu requires “standby diesel.”	Lao PDR. (2) Tuvalu (7)
***N6 Equity, Equality, and Climate Justice*** **N6.1 Inclusiveness**	
Consider gender, youth, and vulnerable groups as cross-cutting issues to be incorporated into disaster risk and vulnerability assessments as well as development and adaptation actions/plans.	Burundi (12) Nepal (8) Timor-Leste (22) Uganda (15)
Interregional socio-economic equality, human rights, and gender equality.	Central AR. (15)
Despite being the smallest contributors to GHG emissions, the Solomon Islands and Kiribati are at the frontline of the wrath of sea-level rise and climate change. These countries consider their NDCs a “moral imperative” as global citizens. They consider themselves to have a “right to develop” their economy and improve the wellbeing of their population.”	Kiribati (27) Solomon I. (21)
***N7 Public Health*** **N7.1 Applied**	
Climate-resilient health facilities, intensive care units for treating heat-related disorders, and interventions for dealing with climate-related health hazards.	Myanmar (45)
“Prevention of waterborne diseases and seasonal pathologies”. Effective elimination or control of COVID-19 transmission.	Central A.R. (11) Solomon I. (21)
**N7.2 Preventive**	
Protect social and economic systems against the vulnerabilities of coastal areas and the rising sea level (and local landscapes). Prepare for any difficult situation which might arise from poor capacity structures and enhance adaptive capacity. Interpret, communicate, and guide local communities against climate change.	Burundi (4) Lesotho (23)

^1^ A non-exhaustive list of references. See Table 1 for a complete overview of each country’s needs.

## Data Availability

Please refer to the NDC registry webpage of the UNFCCC.
